# Seatbelt use and risk of major injuries sustained by vehicle occupants during motor-vehicle crashes: a systematic review and meta-analysis of cohort studies

**DOI:** 10.1186/s12889-018-6280-1

**Published:** 2018-12-29

**Authors:** Nicole Fouda Mbarga, Abdul-Razak Abubakari, Leopold Ndemnge Aminde, Antony R. Morgan

**Affiliations:** 1 Médecins Sans Frontières, Yaounde, Cameroon; 2 School of Health and Life Sciences, Glasgow Caledonian University London, London, UK; 3 Faculty of Medicine, School of Public Health, The University of Queensland, Brisbane, Australia

**Keywords:** Seatbelt, Risk, Injury, Adult, Passengers, Vehicle

## Abstract

**Background:**

In 2004, a World Health Report on road safety called for enforcement of measures such as seatbelt use, effective at minimizing morbidity and mortality caused by road traffic accidents. However, injuries caused by seatbelt use have also been described. Over a decade after publication of the World Health Report on road safety, this study sought to investigate the relationship between seatbelt use and major injuries in belted compared to unbelted passengers.

**Methods:**

Cohort studies published in English language from 2005 to 2018 were retrieved from seven databases. Critical appraisal of studies was carried out using the Scottish Intercollegiate Guidelines Network (SIGN) checklist. Pooled risk of major injuries was assessed using the random effects meta-analytic model. Heterogeneity was quantified using I-squared and Tau-squared statistics. Funnel plots and Egger’s test were used to investigate publication bias. This review is registered in PROSPERO (CRD42015020309).

**Results:**

Eleven studies, all carried out in developed countries were included. Overall, the risk of any major injury was significantly lower in belted passengers compared to unbelted passengers (RR 0.47; 95%CI, 0.29 to 0.80; I^2^ = 99.7; *P* = 0.000). When analysed by crash types, belt use significantly reduced the risk of any injury (RR 0.35; 95%CI, 0.24 to 0.52). Seatbelt use reduces the risk of facial injuries (RR = 0.56, 95% CI = 0.37 to 0.84), abdominal injuries (RR = 0.87; 95% CI = 0.78 to 0.98) and, spinal injuries (RR = 0.56, 95% CI = 0.37 to 0.84). However, we found no statistically significant difference in risk of head injuries (RR = 0.49; 95% CI = 0.22 to 1.08), neck injuries (RR = 0.69: 95%CI 0.07 to 6.44), thoracic injuries (RR 0.96, 95%CI, 0.74 to 1.24), upper limb injuries (RR = 1.05, 95%CI 0.83 to 1.34) and lower limb injuries (RR = 0.77, 95%CI 0.58 to 1.04) between belted and non-belted passengers.

**Conclusion:**

In sum, the risk of most major road traffic injuries is lower in seatbelt users. Findings were inconclusive regarding seatbelt use and susceptibility to thoracic, head and neck injuries during road traffic accidents. Awareness should be raised about the dangers of inadequate seatbelt use. Future research should aim to assess the effects of seatbelt use on major injuries by crash type.

**Electronic supplementary material:**

The online version of this article (10.1186/s12889-018-6280-1) contains supplementary material, which is available to authorized users.

## Background

Globally, the burden of trauma is currently a major public health concern. Road traffic injury (RTI) is the ninth foremost cause of death worldwide [1] and the eighth cause of disability-adjusted life year (DALY) [2]. The average number of deaths occurring on the world’s roads reaches 1.24 million every year [3]. RTIs also cause 20 to 50 million non-fatal injuries yearly followed by disability [3]. Globally, the majority of deaths from road traffic accidents (RTAs) occur in car occupants (31%), followed by motorised two to three wheelers (23%) and pedestrians (22%) [4]. RTIs pose a huge economic burden to countries globally and especially for developing countries where the cost of RTAs represented between 1 and 2% of Gross National Product (GNP) every year [5]. Therefore, the need for effective prevention of RTAs across the world is an imperative.

The World Health Organisation (WHO) published a report in 2004 on interventions effective in addressing and reversing the trends of RTAs [6]. Prominent among these interventions, is the enforcement of seatbelt usage [6]. Non-adherence to seatbelt usage increases the likelihood of being injured during motor vehicle crashes (MVCs) [7]. Moreover, compliance with seatbelt use has been associated with reduced mortality after MVCs in several countries, as well as reduced severity of injuries in car occupants [8–10]. Indeed, since the 1960s, proper usage of seatbelts has been proven effective in reducing crash fatality rates [8, 11]. Crandall et al [12] assessed the efficacy of passenger’s safety equipment during MVCs in the United States (US). They found that the use of lap-shoulder belts was associated with decreased fatality by 72% (OR = 0.28, 95% CI: 0.25, 0.31). Similarly, Dinh- Zar et al [13] demonstrated that the effectiveness of seatbelt use was greater in those accidents with increased likelihood of ejection such as ‘run-off-the-road’ and frontal crashes. Sen and Mizzen [14] in a Canadian study, found that a 1% increased use of seatbelts was associated with between 0.17 to 0.21% reduction in crash fatality. In a more recent study, Cumins and collaborators [9] analysed the US National Trauma Data Bank. They found that the use of seatbelts reduced mortality by 51% for car occupants. This reduction increased to 67% with the combination of seatbelt usage and airbags.

However, research suggests that seatbelt use may also be associated with some adverse outcomes. A plethora of seatbelt injuries are increasingly being described in the literature [15–22]. Signs of seatbelt usage on the body are pathognomonic of seatbelt injuries [10]. A seatbelt sign is a linear skin discoloration secondary to bleeding under the skin caused by seatbelts during MVCs [23]. The seatbelt syndrome refers to an umbrella term used to refer to a variety of injuries caused by seatbelts to car occupants during motor vehicle accidents (MVAs) [24]. The seatbelt syndrome encompasses a seatbelt sign on the chest or the abdominal wall, a fracture of the lumbar section of the spine and perforation of a hollow viscus [17, 23, 25]. Incorrect use of seatbelts in adults has been associated with seatbelt syndrome [19] although it has been demonstrated that the paediatric population is affected most. [26]. Lapbelts usually cause abdominal, pelvic or spinal injuries. In addition to abdominopelvic and spinal injuries, the three-point belt is associated with injuries of the chest, the heart, lungs, the brachial plexus of nerves and major blood vessels [25, 27]. When a seatbelt sign occurs on the abdominal wall, abdominal organs might be injured [28].

In spite of poor outcomes being associated with improper use of seatbelts, it is generally accepted that proper use of seatbelts is effective in reducing fatalities [10]. There remains a gap however, in the literature concerning the effects of seatbelt use on specific major road traffic-related injuries. Given the increasing attention and action on road safety, it is important to ascertain the exact relationship between seatbelt use and major injuries of vehicle occupants during vehicle crashes. This review sought to investigate the association between seatbelt use and the risk of specific major road traffic injuries among adult car occupants. Injuries considered as major road traffic-related injuries in this paper are head injuries, neck injuries, facial injuries, spinal injuries, thoracic injuries, abdominal injuries and limb injuries.

## Methods

Details of the protocol for this review were registered at the Centre for Reviews and Dissemination database in May 2015 (see doi 10.15124/CRD42015020309 in http://www.crd.york.ac.uk/PROSPERO/display_record.php?ID=CRD42015020309). Relevant elements of the Meta-analysis of Observational Studies in Epidemiology (MOOSE) guidelines were followed in conducting and reporting the findings of this review [29].

### Search strategy

Searches were conducted by two independent reviewers in electronic databases, through citation trails and references of key papers. Databases searched included Web of Science (all databases), Science direct, Springer link, Biomed central, Embase, the EBSCO host and all regional indexes of the Global Index Medicus (GIM). Relevant Boolean operators (AND, OR, NOT) were used to combine appropriate Medical Subject Headings (Mesh) and/or keywords.

Key words and associated search terms used were combined as depicted in the following search equation: Σ = [(seatbelt OR lapbelt OR shoulder belt OR safety belt OR car restraint OR three point belt), AND (impact OR effect) AND {(spinal injuries OR spinal cord injuries OR spinal fractures OR spinal cord compression); OR (abdominal injuries OR liver injuries OR hepatic injuries OR splenic injuries OR kidney injuries OR intestinal injuries), OR (thoracic injuries OR cardiac injuries OR chest injuries OR lung injuries OR pulmonary injuries OR heart injuries), OR (facial injuries OR mandibular fractures OR maxillofacial injuries OR orbital fractures OR corneal injuries OR skull fractures OR mandibular injuries OR eye injuries OR jaw fractures), OR (head injuries OR brain injuries OR cerebral hemorrhage OR brain concussion OR craniocerebral trauma OR neck injuries), OR (limb injuries OR lower extremity injuries OR upper extremity injuries)}, AND (adults OR occupants OR passengers OR drivers OR seaters) NOT (children OR paediatric OR infants OR pregnant)]

Study selection was independently carried out by two coinvestigators. Disagreements were resolved by consensus. Titles and abstracts of all papers retrieved from the searches were screened for selection using predefined inclusion/exclusion criteria. This was followed by retrieval of full texts of articles which met the inclusion criteria. At this stageStudies meeting one or more exclusion criteria were not included. Reference lists of included studies were searched for additional studies which might have been missed by our searches. Initial search and selection of articles were carried out between June and august 2015 and updated in July 2018

### Inclusion/exclusion criteria

Articles reporting seatbelt use and injury outcomes were considered. Given that cohort studies provide stronger evidence than case control studies [30], only cohort studies published in English from January 2005 (after publication of the world health report on road safety in 2004) to July 2018 and comparing adult seatbelt users and non-users were considered for selection. Subsequently, studies were categorized into relevant major injuries in line with our research objectives. Selection of studies was not based on specific types of seatbelt or vehicle or crash type. Thus, all forms of seatbelts, all types of MVCs and all types of collisions were considered. Vehicles included cars, vans or trucks. Outcomes of interest were types of injury: head, neck, facial, thoracic, abdominal, spinal, and limb injuries.

### Data extraction and quality assessment

Quality assessment was carried out using the Scottish Intercollegiate Guidelines Network (SIGN) tool for cohort studies [31]. The SIGN tool for cohort studies has 18 items: five items address issues in the selection of study participants; four items assess measurement of outcomes while two address measurement of exposure; four items address other forms of biases; two items assess the control of confounding; and one item assesses statistical analysis [31]. The SIGN tool also provides an overall quality score for studies. Low quality studies which had at least three negative answers for the relevant items listed above were rejected based on lack of appropriate methodological robustness. Studies included after the methodological quality appraisal were categorised into acceptable or highly acceptable. Data extracted from included studies were: study characteristics (title, authors, and references), methods (sampling and sample size, assessment of bias) and findings relevant to this review (e.g. seatbelt use and injury outcomes).

### Meta-analysis

As this review included observational studies (cohort design), it was appropriate to assume variations in effect size between individual studies, thus the random effects model was used for statistical synthesis. Pooled relative risk and confidence intervals were calculated and where possible, prediction intervals for the pooled effect sizes were estimated. Tests for statistical heterogeneity including I^2^, τ^2^ and H statistics were computed to examine potential variability of the effect estimate between the studies beyond the effect of chance. Meta-analyses were conducted by crash type, and separately for the different anatomical categories of road traffic-related injuries: head injuries, neck injuries, facial injuries, spinal injuries, thoracic injuries, abdominal injuries and limb injuries. Where possible, subgroup analysis was conducted to explore potential sources of heterogeneity. The funnel plot and the Egger’s regression test were used to detect evidence of publication bias. STATA version 15, StataCorp, College Station, TX, USA was used for statistical analysis.

## Results

### Description of included studies

**Fig. 1:**
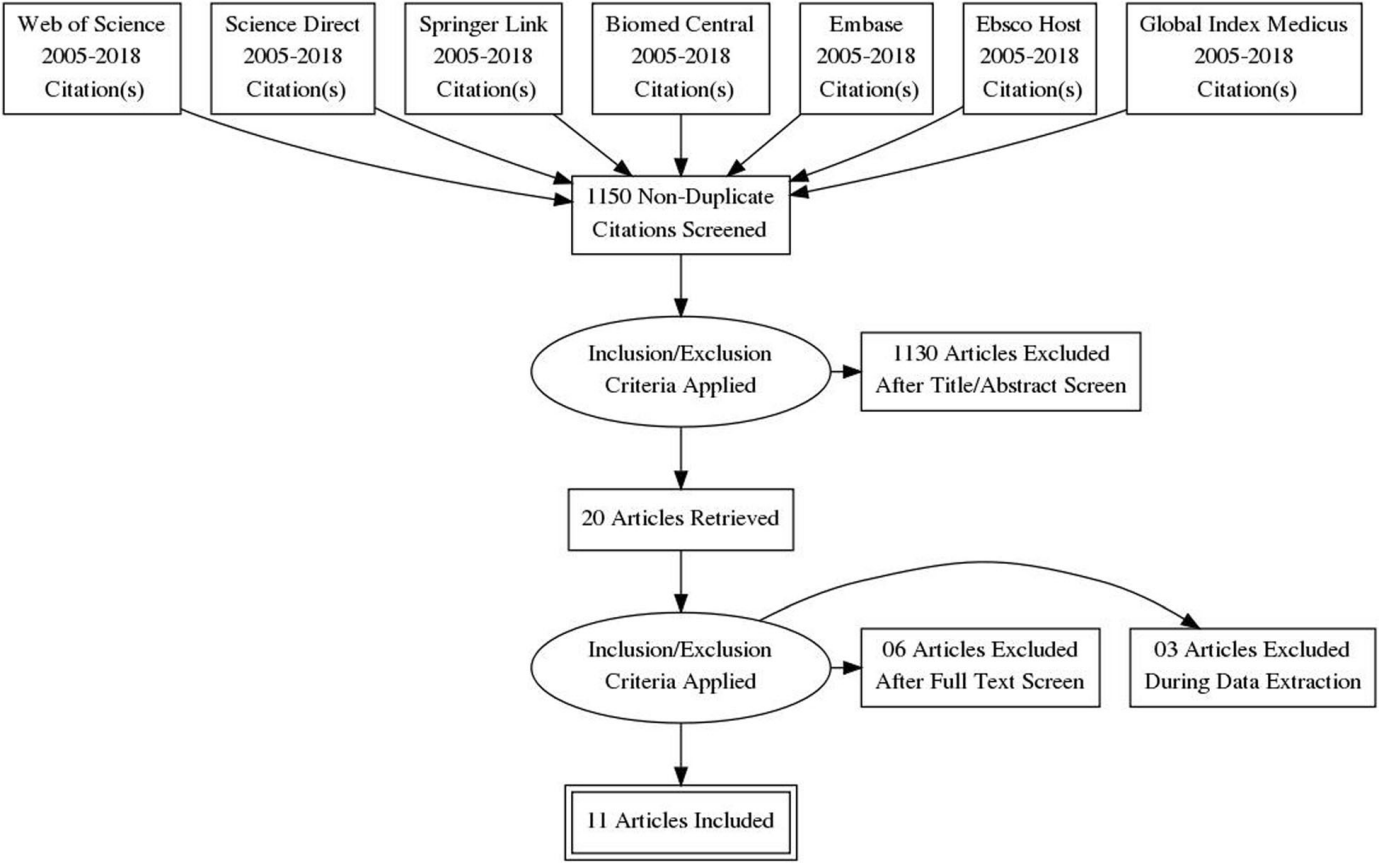
Flow diagram of retrieved searches

The total number of records identified through the literature search process in all databases after removal of duplicates was 1150 (Fig. 1). Searches conducted from reference lists of key articles and citation trails did not yield results. Titles and abstract were screened. Finally, full texts were screened and only 11 papers were included in the meta-analysis [32–42]. Three studies included were conducted in hospital settings - two of which were conducted in Tampa hospital, Florida, United States (US) and one in Tawan Hospital, United Arab Emirates (UAE) [35, 40, 41]. The remaining eight studies were based on passenger information retrieved from databases in Canada, the United States and Australia [32–39, 42]. Databases used for these studies included: the National Automotive Sampling System- Crash Worthiness Data System (NASS-CDS), Crash Injury Research and Engineering Network (CIREN) and the National Trauma Data bank (NTDB) for US data; the Office of the Chief Coroner of Ontario (OCCO) for Canadian records; and the Australian National Coronial Information System (NCIS). Sample sizes across studies ranged from 22 [41] to 41,596,417 participants [32]. All 11 studies [32–42] involved individuals aged 15 years and above. Four studies [35, 39–41] focused on middle-aged individuals (mean age < 40 years), see Additional file 1.

### Risk of any major injuries in belted versus unbelted passengers

**Fig. 2:**
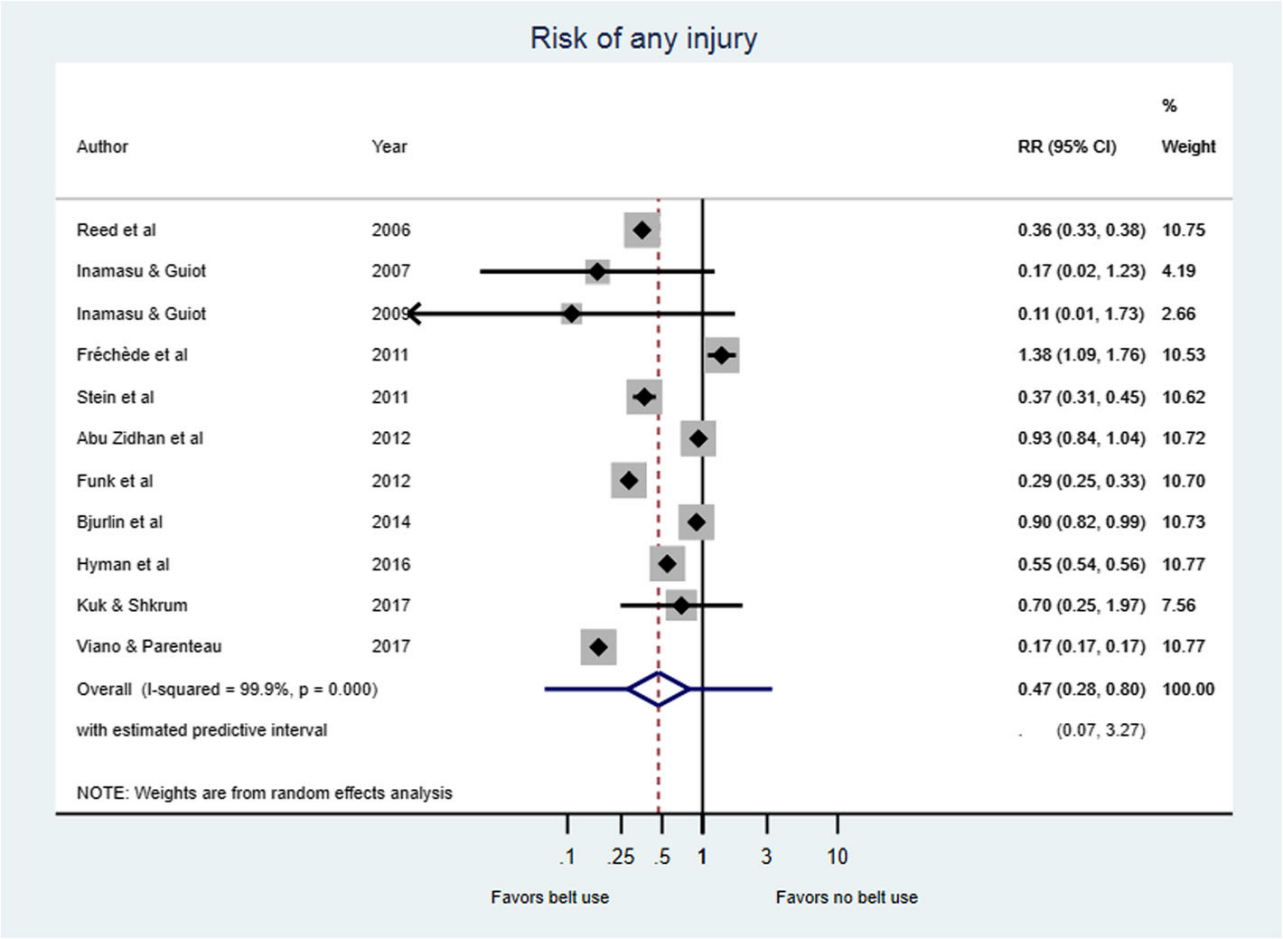
Risk of any injury in belt versus non-belt users. On the forest plots, black diamonds represent individual risk of injury (by study); Blue diamonds represent the subtotal of risk of injury by subcategory; Black lines represent the individual 95% confidence intervals; Blue lines represent the estimated predictive intervals; Blue dotted lines represent the inestimable predictive intervals

**Fig. 3:**
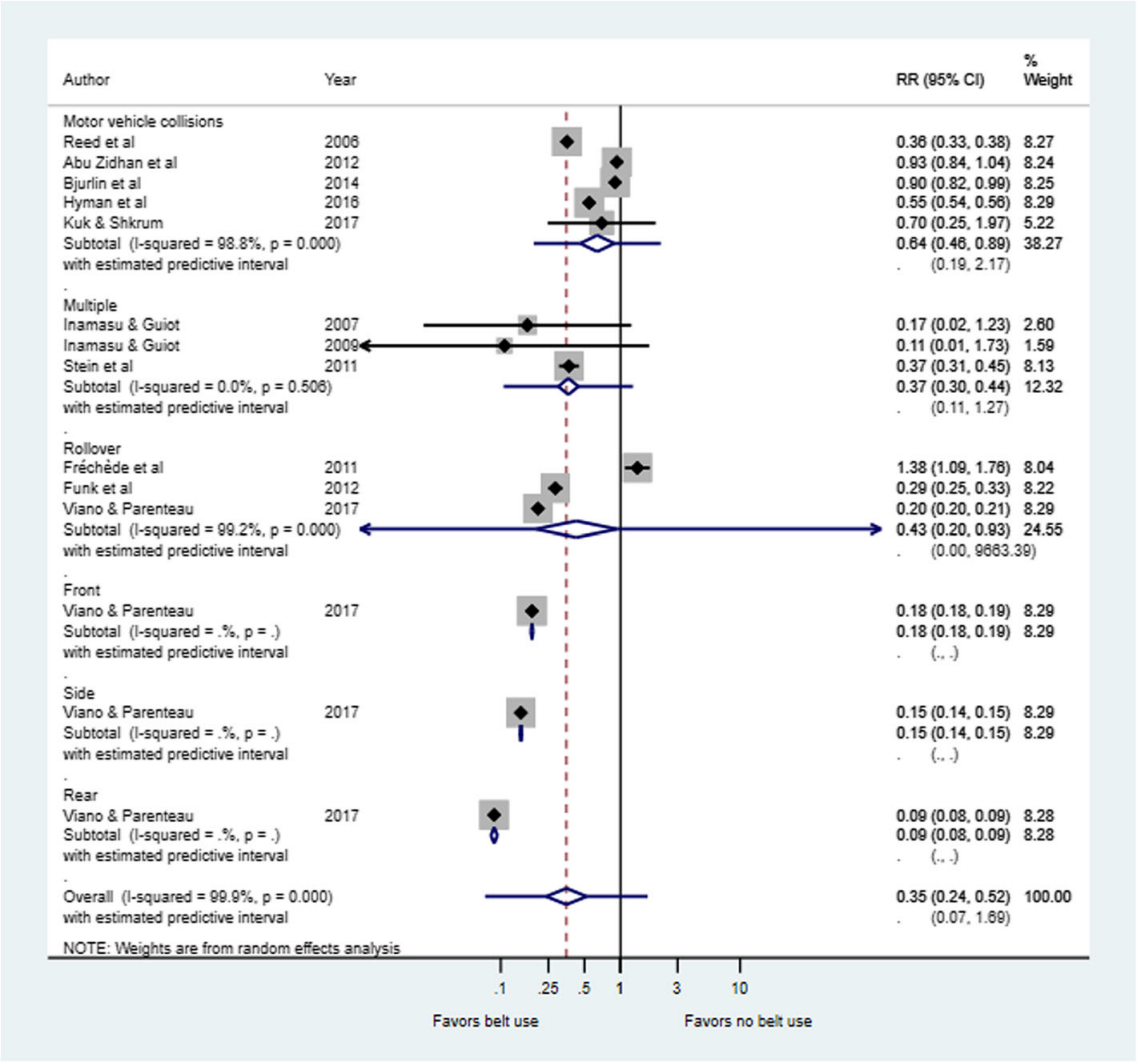
Risk of any injury by crash type in belt users versus non-belt users. On the forest plots, black diamonds represent individual risk of injury (by study); Blue diamonds represent the subtotal of risk of injury by subcategory; Black lines represent the individual 95% confidence intervals; Blue lines represent the estimated predictive intervals; Blue dotted lines represent the inestimable predictive intervals

The use of seatbelts was associated with lower risk of major injuries in six [32, 34, 35, 38, 39, 42] of the 11 studies included in the meta-analysis. However, the level of risk reduced as a result of wearing a seatbelt varied notably across studies (Fig. 2). Overall, the risk of any major injury was significantly lower in seatbelt users compared to non-users as shown in the combined relative risk in Fig. 2 (RR = 0.47, 95%CI: 0.28 to 0.80; Tau^2^ = 0.669; I^2=^99.9; *p* < 0.0001). Figure 3 shows that passengers using seatbelts also had significantly lower risk of injury compared to unbelted passengers when analysis was conducted by crash type (RR = 0.35; 95%CI, 0.24 to 0.52; I^2^ = 99.9%; *P* = 0.000). Table 1 presents a summary of the effect estimate in various subgroups.

**Table 1:** Comparison of effect estimate by various subgroups

Grouping variable	Subgroup	Numb. of studies	Sample	RR (95% CI)	Prediction intervals	Tau^2^	I^2^ (95%CI)	H (95%CI)	*P*-value for heterogeneity	*P*-value Egger’s test	*P*-value difference in subgroups
Overall (any injury)	**–**	11	38,662,538	0.47 (0.28–0.80)	0.07–3.27	0.669	99.9 (99.9–99.9)	34.7 (33.2–36.4)	< 0.0001	0.361	NA
By injury site	Head	6	32,964,587	0.49 (0.22–1.08)	0.03–8.65	0.910	99.3 (99.1–99.4)	11.6 (10.1–13.4)	< 0.0001	0.046	0.090
	Spinal	7	5,281,081	0.56 (0.37–0.84)	0.15–2.01	0.206	93.1 (88–96)	3.8 (2.9–5.0)	< 0.0001	0.328	
	Facial	2	409,016	0.75 (0.40–1.43)	–	0.207	96.9	**–**	< 0.0001	**–**	
	Neck	2	855	0.69 (0.07–6.44)	–	1.761	56.6	**–**	0.129	**–**	
	Thoracic	2	855	0.96 (0.74–1.25)	–	0.0	0.0	**–**	0.754	**–**	
	Abdomen	2	4621	0.93 (0.84–1.04)	–	0.0	0.0	**–**	0.912	**–**	
	Upper limb	1	766	1.05 (0.83–1.34)	**–**	**–**	**–**	**–**	**–**	**–**	
	Lower limb	1	766	0.77 (0.58–1.04)	**–**	**–**	**–**	**–**	**–**	**–**	
By crash type	Motor vehicle collision	5	5,692,713	0.64 (0.46–0.89)	0.19–2.17	0.119	98.8 (98–99)	9.0 (7.4–10.8)	< 0.0001	0.670	0.001
	Rollover	3	3,276,677	0.43 (0.20–0.93)	0.0–963.4	0.465	99.2 (99–99.6)	11.4 (9.1–14.3)	< 0.0001	0.268	
	Multiple	3	3447	0.37 (0.30–0.44)	0.11–1.27	0.0	0.0 (0.0–90)	1.0 (1.0–3.1)	0.506	0.025	
	Front	1	18,296,847	0.18 (0.18–0.19)	**–**	**–**	**–**	**–**	**–**	–	
	Side	1	8,571,748	0.15 (0.14–0.15)	**–**	**–**	**–**	**–**	**–**	**–**	
	Rear	1	2,821,106	0.09 (0.08–0.09)	**–**	**–**	**–**	**–**	**–**	**–**	
By study sample size	< median (6128)	5	4662	0.51 (0.20–1.30)	0.02–12.46	0.783	94.5 (90–97)	4.3 (3.2–5.8)	< 0.0001	0.121	0.594
	≥ median (6128)	6	38,657,876	0.45 (0.23–0.86)	0.04–5.10	0.659	99.9 (99.9–99.9)	48.7 (46.3–51.2)	< 0.0001	0.340	
By study type	Hospital	3	6189	0.39 (0.01–1.72)	0.0–5025	1.079	61.1 (0–89)	1.6 (1.0–3.0)	0.077	0.038	0.967
	Patient databases	8	38,656,349	0.48 (0.27–0.84)	0.06–3.88	0.648	99.9 (99.9–99.9)	40.6 (38.6–42.6)	< 0.0001	0.452	
Publication year	Before 2010	3	5,270,440	0.36 (0.33–0.38)	0.22–0.57	0.0	0.0 (0.0–90)	1.0 (1.0–3.1)	0.530	0.036	0.269
	On or after 2010	8	33,392,098	0.54 (0.30–0.98)	0.06–4.75	0.697	99.9 (99.9–99.9)	41.4 (39.4–43.4)	< 0.0001	0.388	

NB: Absent predictive intervals could not be estimated due to inadequate number of studies (less than 3); absent I^2^ confidence intervals could not be estimated due to degrees of freedom less than 2 and Egger’s test was inestimable for groups with less than 3 studies

### Risk of spinal injuries

**Fig. 4:**
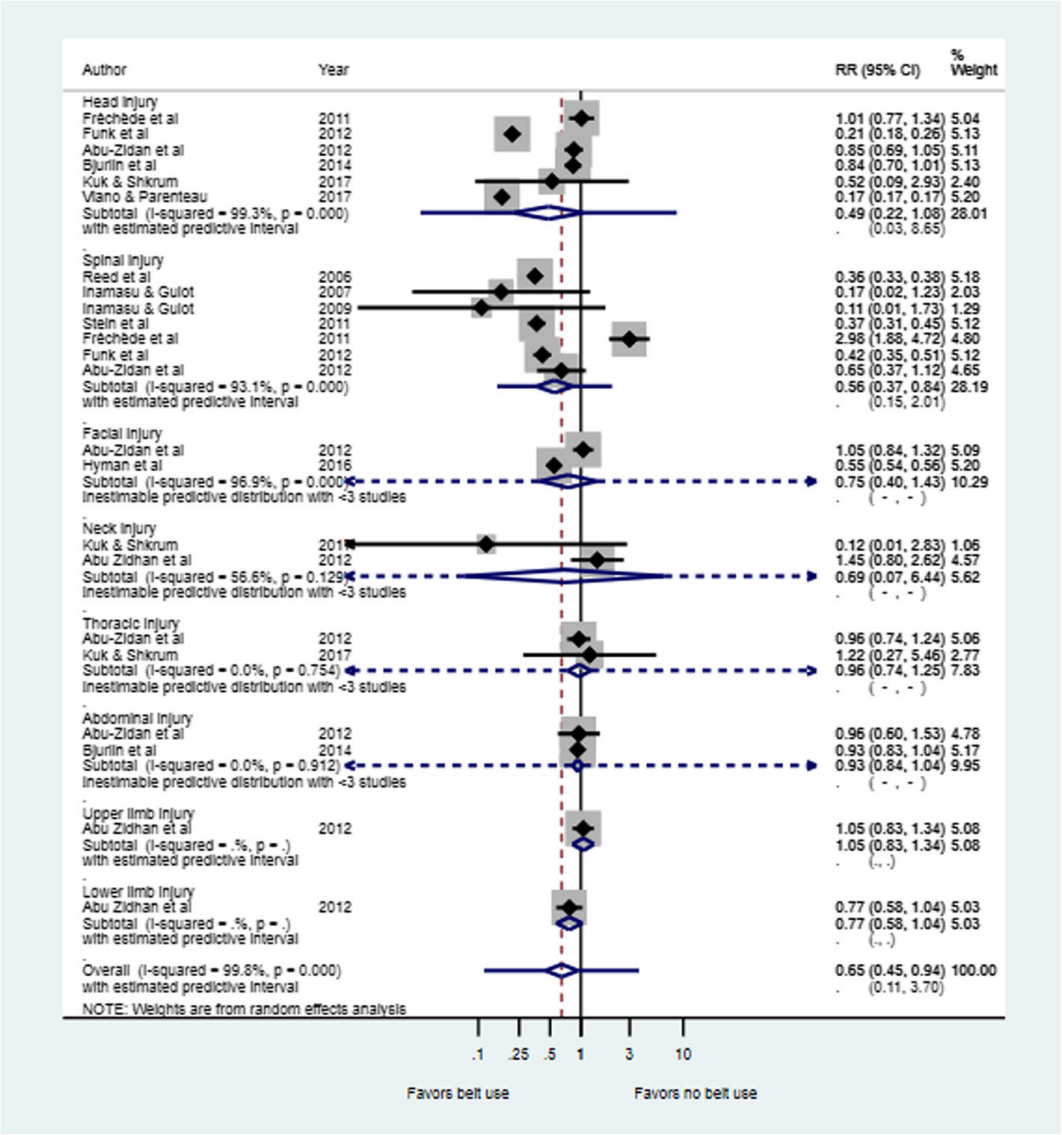
Risk by major body region injury in seatbelt users versus non-belt users. On the forest plots, black diamonds represent individual risk of injury (by study); Blue diamonds represent the subtotal of risk of injury by subcategory; Black lines represent the individual 95% confidence intervals; Blue lines represent the estimated predictive intervals; Blue dotted lines represent the inestimable predictive intervals

Figure 4 is a forest plot depicting the risk of injury by major body region in belt users compared to non-belt users. Risk ratios of spinal injuries varied from 0.11 (95% CI: 0.01 to 1.73 in Inamasu & Guiot [41] to 2.98 (95%CI = 1.88 to 4.72) in Fréchède et al [33] as shown in Fig. 4. The pooled risk ratio of spinal injury was 0.56 (95% CI = 0.37 to 0.84) with a high degree of heterogeneity across studies (Tau^2^ = 0.206; I^2^ = 93.1%, *P* < 0.0001) (Table 1).

### Risk of head injuries

As shown in Fig. 4, there was no statistically significant difference in risk of head injury when pooled analysis was conducted (RR for pooled analysis =0.49; 95% CI = 0.22 to 1.08). Investigations for heterogeneity between the studies included in this pooled analysis showed a statistically significant high heterogeneity between study results (Tau^2^ = 0.910; I^2^ = 99.3%; *P* < 0.0001) (Table 1).

### Risk of facial injuries

Two studies reported data on facial injuries in relation to seatbelt use [35, 36](Fig. 4). Taken together there was no statistically significant difference in risk of facial injury between seatbelt users compared with passengers who did not use seatbelts (RR = 0.75, 95%CI: 0.40 to 1.43), I^2^ = 96.9%; *p* < 0.0001).

### Risk of thoracic injuries

Two studies reported data on thoracic injuries [35, 37] as seen in Fig. 4. The pooled risk ratio for thoracic injury also showed no statistically significant difference in risk of thoracic injury between seatbelt users and non-users (RR = 0.96, 95%CI: 0.74 to 1.24; Tau^2^ = 0.0, I^2^ = 0%, *p* = 0.754).

### Risk of abdominal injuries

Two studies reported data on abdominal injuries [35, 39]. The pooled risk ratio showed significantly lower risk of abdominal injury in seatbelt users compared to non-users (RR = 0.87, 95%CI: 0.78 to 0.98, I^2^ = 0%; *p* = 0.912) (Fig. 4).

### Risk of neck injuries

There was no statistically significant difference in risk of neck injury between belted and non-belted passengers as shown in Fig. 4. The pooled estimate showed there was a non-significant effect (RR = 0.69, 95%CI: 0.07 to 6.44, Tau^2^ = 1.761; I^2^ = 56.6%, *p* = 0.129) (Fig. 4).

### Risk of limb injury

Abu Zidhan et al [35] reported no statistically significant difference in risk of upper limb injury (RR = 1.05, 95%CI: 0.83 to 1.34) and risk of lower limb injury (RR = 0.77, 95%CI: 0.58 to 1.04) in belted versus non-belted passengers. Heterogeneity was inestimable due to the fewer number of studies included (Fig. 4).

### Subgroup analyses

In addition to assessing effect estimates by major anatomical injury site and crash type, other potential sources of heterogeneity that might impact the effect estimate for the risk of any injury across the studies were explored. These were: sample size (at or above the median versus below median); publication date (before 2010 versus during or after 2010); study type (hospital-based versus patient information databases). This subgroup analysis showed neither substantive heterogeneity within different characteristics nor statistically significant differences within groups (Table 1).

### Publication bias

**Fig. 5:**
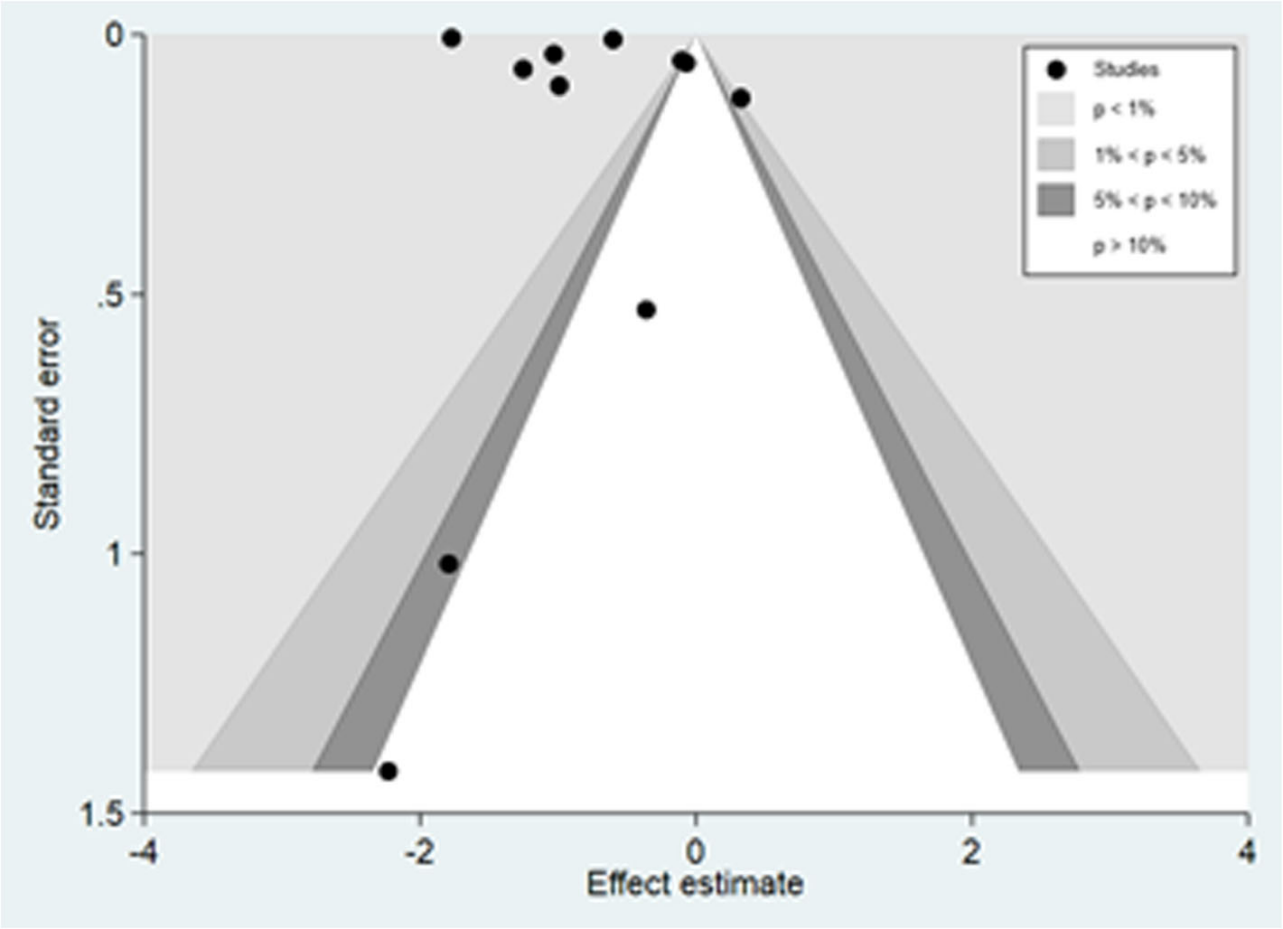
Contour-enhanced funnel plot for publication bias

The funnel plot suggested some asymmetry and therefore on first observation some publication bias was inferred. However given the contour-enhanced feature also observed and the small study samples included in the review, there is some indication that the asymmetry in the funnel plot could be due to factors other than publication bias (Fig. 5). For instance, in comparisons where there is no intervention or exposure effect, publications influenced by the *p* value alone (e.g. the tendency to publish extreme negative or positive findings compared to no effect) may lead to asymmetrical funnel plot. Moreover, the fact that some measures of effect are highly correlated with their standard errors (e.g. odds ratios) may lead to spurious asymmetrical funnel plot [43]. The subsequent Egger’s regression test suggested no evidence for publication bias (*p* = 0.361) overall. However when the Egger’s test was carried out on the effect estimate in the subgroup analysis, there was some evidence of publication bias. Specifically, for reports on head injuries (*p* = 0.046), studies involving multiple crash types (*p* = 0.025), studies conducted in hospital (*p* = 0.038) and studies published before 2010 (*p* = 0.038) (See Table 1).

## Discussion

This systematic review and meta-analysis carried out to assess the associations between seatbelt use and major injuries of vehicle occupants during RTAs appears to be the first of its kind. Overall, seatbelt use has been shown to provide a 53% reduction in risk of any injury among vehicle occupants following MVAs. However, this review suggests that seatbelt use has a more limited safety when specific outcomes are studied including head injuries, neck injuries, thoracic injuries and limb injuries. The pooled estimate for risk of injury by crash types also varied. Taken together this review was not able to provide evidence to demonstrate the protective role of seatbelt use for these outcomes.

Importantly however, findings from this review indicate that seatbelt use reduces the risk of facial injuries, spinal injuries, and abdominal injuries. By and large, there was no statistically significant heterogeneity between studies included in the pooled estimate for neck, thoracic and abdominal injuries. Perhaps, this is due to the fact that only 2 studies were used in quantifying the risk of these injuries. However, there was a significantly high heterogeneity in the effect estimates of the studies in the pooled analysis for facial injuries, head injuries as well as for spinal injuries. One of the studies included in the pooled analysis for risk of head and spinal injuries focused on car rollover fatalities [33] whereas the other studies analyzed MVCs in general, be it fatalities or not. Perhaps this could also explain the high heterogeneity.

Our findings concur with previous studies on this topic. For instance, Hilary et al [44] found no relationship between seatbelt use and severity of head injury (*P* = 0.13) and that unbelted car occupants are more prone to posterior brain lesions. These findings support the inability of our study to demonstrate the protective role of seatbelts against head injuries. On the contrary, it has also been demonstrated in some case series that seatbelt use is associated with reduced severity of brain injury and decreased incidence of brain injuries [45, 46]. Similarly, Mohammadzadeh et al [47] observed that seatbelt use reduces the proportions of head injury. Thus, evidence on the impact of seatbelt use on head injuries seems to be inconclusive.

The protective role of seatbelts against spinal injuries, and abdominal injuries also seems equivocal given that seatbelt injuries have been reported. Although findings of the current study suggest that the risk of abdominal injuries is lowest for belted occupants, the seatbelt syndrome which encompasses abdominal injury, has been extensively described in the literature as caused by improper seatbelt use [17, 23, 25]. This syndrome mostly affects the pediatric population [26], but it has also been documented in adults [22]. Improper seatbelt use has also been associated with intra-abdominal and spinal injuries [16, 22]. Seatbelt use has been shown to be associated with increased spinal injury severity, neurological deficit and fatality [48]. Compared to unbelted individuals, the frequency of thoracic and lumbar spinal injuries was shown to be higher in belted occupants [48].

It is important to note however, that the majority of evidence not supporting the protective role of seatbelt use against major injuries of car passengers stems from case-series, case-reports and some few cross-sectional and case-control studies [43, 45–47]. These study designs are prone to numerous forms of bias and confounding, and therefore not methodologically robust enough to infer causality in the relationships between exposure and outcomes [49]. Findings from such studies therefore need to be interpreted with caution.

It has been observed that seatbelt injuries vary with crash types. For instance, Kuan et al [50] observed that renal injuries associated with seatbelt use mostly occurred in frontal and side impact collisions. In these instances, drivers usually suffer from right kidney injuries, while left renal injuries were characteristics of other car occupants. Dinh- Zar and colleagues [13] reported that, perhaps, the protective effect of seatbelt use is more pronounced during frontal crashes as they have higher likelihood of ejection during the RTAs such as run-off-the-road collision. Indeed our study found significant differences in risk of major injury between users and non-users of seatbelt by crash types as shown in Fig. 3. In most cases: including multiple, roll over, side or rear, risk of injury was lower among passengers who wore a seatbelt compared to those who did not. However, data for some crash types (front, side and rear) were provided by only one study each which would suggest further investigations are required before firmer conclusions can be made.

It should be noted that other studies do exist that might improve our knowledge in this area but were excluded from our review on the basis of exclusion criteria. For instance, Ogundele and collaborators assessed the impact of seatbelt usage in limiting injury severity during MVCs [51] but was excluded as it included a paediatric population for whom special restraints are used. More recently, Renson et al. described the relationships between seatbelt use and risk of high grade hepatic injury [52]. Similarly, Mc Mullin et al. analysed facial injuries in relationship with seatbelt use [53]. Such papers were rejected as only patients with specific injuries were included (no comparison group), thus estimation of risk of injuries in belted versus unbelted car occupants was not possible. Matsui and collaborators recently described factors causing abdominal injuries during frontal collisions [54]. This was a simulation using a dummy wearing a three point seatbelt and for this reason, this experimental study was excluded. Lastly, some studies with less robust methodology (case-series, case-studies and cross sectional studies) [46, 47, 49] were excluded due to the inherent bias which would have been introduced.

This review synthesized the best available evidence from cohort studies on seatbelt use and road traffic injuries. Despite filling an important gap in the literature, our study does have limitations. Like most observational studies, meta-analysis of cohort studies is less methodologically robust compared to meta-analysis of well-conducted randomized controlled trials (RCTs) [55]. Thus, inclusion of observational studies only might have distorted effect estimates potentially contributing to the high heterogeneity found in this study. In addition, ten of the studies included [32–42] in this review used secondary data to investigate the effectiveness of seatbelt use. It is difficult to determine the adequacy of selection of the study population, measurements of exposure and outcomes in retrospective cohort studies. Moreover, the methodological quality appraisal conducted as part of this review rated all eleven studies included as acceptable in strength indicating some inherent weaknesses in the studies included in this review. In addition, due to the limited number of studies included, multivariable meta-regression could not be performed to evaluate the impact of potential covariates, which might explain heterogeneity. Thus, exploration of heterogeneity was not exhaustive. Additionally, only studies published in English were included in the review, thus the potential for language bias cannot be ruled out. Lastly, data was not available to ascertain if seatbelt use was in conformity with the current legislation. In the pooled analyses and where possible, prediction intervals were estimated to better make sense out of estimates given the large variations. Although we found a beneficial effect of seatbelt use (for risk of any injury and some injury types and crash types), prediction intervals in most instances suggested that future studies might include the null or opposite effect of seatbelt use on risk of injury. This would however pertain specifically to studies conducted in similar populations or settings as those included in this meta-analysis. The transferability of the impact of future studies conducted in other settings (different from those in our meta-analysis) might potentially be different and not necessarily contradict our findings as acknowledged by other authors [56]. Future reviews should also consider the possibility of using more inclusive/wider inclusion criteria including studies conducted using other types of observational study designs.

## Conclusions

Overall, the evidence suggests that use of seatbelts reduces the risk of some specific types of injury during road traffic accidents. However, the extent of protection offered by use of seatbelts may be context-specific and public health guidelines on road traffic accidents should be updated to reflect this relative protection conferred by seatbelt usage during MVAs. Further research is required to understand the impact of seatbelt use by crash types, using more representative samples and reliable studies.

## Additional file

Additional file 1: **Table S1.** Study characteristics and outcomes by year of publication. (DOCX 32 kb)

## Abbreviations

AIS: Abbreviated injury scale
CI: Confidence interval
CIREN: Crash injury research and engineering network
DALY: Disability adjusted life years
FIA: Foundation for the automobile and society
GCS: Glasgow Coma Score
GIM: Global Index Medicus
GNP: Gross national product
ISS: Injury severity score
MeSH: Medical subject heading
MVAs: Motor vehicle accidents
MVCs: Motor vehicle crashes
NASS-CDS: National automotive sampling systems- Crash worthiness data system
NCIS: National coronial information system
NTDB: National trauma data bank
OR: Odd ratios
RCTs: Randomised Controlled Trials
RR: Risk ratio
RTAs: Road traffic accidents
RTIs: Road traffic injuries
SIGN: Scottish intercollegiate guideline network
US: United States
WHO: World Health Organisation
