# Supplementary material for: Seatbelt use and risk of major injuries sustained by vehicle occupants during motor-vehicle crashes: a systematic review and meta-analysis of cohort studies
Source: BMC Public Health. 2018 Dec 29;18:1413. doi: 10.1186/s12889-018-6280-1 (PMC6310927; doi:10.1186/s12889-018-6280-1)
Supplement: Supplementary file 1 — Table S1. Study characteristics and outcomes by year of publication. (DOCX 32 kb) [file 12889_2018_6280_MOESM1_ESM.docx]

| **Table S1: Study characteristics and outcomes by year of publication.** | | | | | | | | | | | | |
| --- | --- | --- | --- | --- | --- | --- | --- | --- | --- | --- | --- | --- |
| **Study Characteristics:**  **Author**  **Year**  **Setting**  **Country** | **Crash type** | **Participant characteristics** | | | | **Cohort** | | **Outcomes** | | | | |
|  |  | **Age range or mean age (Years)** | **Gender** | | **Sample size** | **Belted** | **Unbelted** | **Type of injury** | | | | **Risk Ratio (RR)** |
|  |  |  | **Number of males** | **Number of females** |  |  |  |  |  |  |  |  |
| **Reed *et al***  **2006**  **NASS-CDS database**  **USA** | Motor vehicle collisions | 16 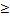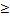 | NR | NR | 8,203,587 (Weighted) | 3,979,566 | 1,290,679 | Spinal injury (CSI) | | | | |
|  |  |  |  |  |  |  |  | Belted Total 1475 | | Unbelted  Total= 1341 | RR= 0.36 (0.33, 0.38) | |
|  |  |  |  |  |  |  |  | AIS 1  N=1425 | | AIS 1  N=1083 |  |  |
|  |  |  |  |  |  |  |  | AIS 2  N=35 | | AIS 2  N=210 |  |  |
|  |  |  |  |  |  |  |  | AIS 3  Total=15 | | AIS 3  Total=48 |  |  |
| **Inamasu & Guiot**  **2007**  **Tampa hospital-USA**  **USA** | Frontal and side rear collision | Belted  37.4 | 28 | 11 | 39 | 18 | 21 | Thoracolumbar junction injury (spinal injury) | | | 0.17 (0.02 -1.23) | |
|  |  |  |  |  |  |  |  | Belted | | Unbelted |  |  |
|  |  | Unbelted  28 |  |  |  |  |  | Total= 18 | | Total= 21 |  |  |
|  |  |  |  |  |  |  |  | Neurological deficit N= 1 | | Neurological deficit N= 7 |  |  |
| **Inamasu & Guiot**  **2009**  **Tampa Hospital- USA** | Rollover and side rear collision | Belted  36.3 | 14 | 8 | 22 | 10 | 12 | Thoracolumbar junction injury (spinal injury) | | | 0.11 (0.001-1.73) | |
|  |  | Unbelted  31.8 |  |  |  |  |  | Belted Total= 10 | | Unbelted Total= 12 |  |  |
|  |  |  |  |  |  |  |  | Neurologic deficit N= 0 | | Neurologic deficit N= 5 |  |  |
| **Stein *et al***  **2011**  **NASS CDS/CIREN**  **US** | Frontal, lateral, roolover and other | ≥ 15 | 983 | 1,741 | 2,724 | 2,181 | 600 | Cervical spinal injuries | | | RR= 0.37 (0.31, 0.45) | |
|  |  |  |  |  |  |  |  | Belted Total=145 | | Unbelted Total= 245 |  |  |
| **Fréchède *et al***  **2011**  **NCIS**  **Australia** | Single vehicle rollover | NR | NR | NR | 474 | 80 | 394 | Head injury | | | RR= 1.01 (0.77-1.34) | |
|  |  |  |  |  |  |  |  | Belted N=34 | | Unbelted N=150 |  |  |
|  |  |  |  |  |  |  |  | Spinal injury | | | RR=2.98 (1.88,4.72) | |
|  |  |  |  |  |  |  |  | Belted N=23 | | Unbelted N=58 |  |  |
| **Funk *et al***  **2012**  **NASS-CDS database**  **USA** | Roll over crashes | NR | NR | NR | 6,015 | 3,656 | 2,359 | Head injury (AIS3) | | | RR=0.22 (0.18, 0.26) | |
|  |  |  |  |  |  |  |  | Belted N= 148 | | Unbelted N= 436 |  |  |
|  |  |  |  |  |  |  |  | Cervical Spinal injury | | | RR= 0.42 (0.35, 0.51) | |
|  |  |  |  |  |  |  |  | Belted  N= 154 | | Unbelted N= 236 |  |  |
| **Abu-Zidan *et al***  **2012**  **Tawan hospital**  **UAE** | Motor vehicle collisions | NR | Belted 118 | Belted 17 | 766 | 135 | 631 | Head injuries | | | RR= 0.85 (0.69, 1.04) | |
|  |  |  |  |  |  |  |  | Belted N= 59 | | Unbelted N= 323 |  |  |
|  |  | Belted  34. 2 | Unbelted 478 | Unbelted 153 |  |  |  | Face injuries | | | RR=1.05 (0.84, 1.32) | |
|  |  |  |  |  |  |  |  | Belted N= 55 | | Unbelted N= 323 |  |  |
|  |  |  |  |  |  |  |  | Neck injuries | | | RR= 1.45 (0.83, 2.62) | |
|  |  |  |  |  |  |  |  | Belted N= 13 | | Unbelted N= 42 |  |  |
|  |  |  |  |  |  |  |  | Thoracic injury | | | RR= 0.96(0.74, 1.24) | |
|  |  |  |  |  |  |  |  | Belted N= 45 | | Unbelted N= 220 |  |  |
|  |  |  |  |  |  |  |  | Abdominal injuries | | | RR= 0.96 (0.59, 1.51) | |
|  |  |  |  |  |  |  |  | Belted N= 18 | | Unbelted N= 88 |  |  |
|  |  | Unbelted  28 |  |  |  |  |  | Upper limb injury | | | RR=1.05 (0.84, 1.34) | |
|  |  |  |  |  |  |  |  | Lower limb injury | | | RR= (0.58, 1.04) | |
|  |  |  |  |  |  |  |  | Spinal injuries | | | RR= 0.65 (0.37, 1.10) | |
|  |  |  |  |  |  |  |  | Belted N= 13 | | Unbelted N= 94 |  |  |
| **Bjurlin *et al***  **2014**  **NTDB database**  **US** | Road traffic collisions | Mean age 36.5 | 1741 | 2105 | 3,846 | 1,012 | 1,752 | Head injury AIS>3 | | | RR= 0.84 (0.70, 1.01) | |
|  |  |  |  |  |  |  |  | Belted N= 127 | | Unbelted N= 304 |  |  |
|  |  |  |  |  |  |  |  | Abdomen AIS> 3 | | | RR=0.93 (0.83, 1.34) | |
|  |  |  |  |  |  |  |  | Belted N=288 | | Unbelted N= 574 |  |  |
|  |  |  |  |  |  |  |  | Renal injuries (All grades) | | |  | |
|  |  |  |  |  |  |  |  | Belted N= 404 | | Unbelted N= 790 |  |  |
| **Hyman et al**  **2016**  **NTDB database**  **US** | Motor vehicle collisions | >18 | 302653 | 215485 | 518106 | 187846 | 246760 | Facial injury | | | RR=0.55 (0.54, 0.56) | |
|  |  |  |  |  |  |  |  | Belted  15177 | Unbelted  35771 | |  |  |
| **Kuk & Shkrum**  **2017**  **OCCO**  **Canada** | NR | Mean Age 48.3 | 82 | 28 | 110 | 75 | 28 | Head Injury | | | RR=0.52 (0.09, 2,93) | |
|  |  |  |  |  |  |  |  | Belted N= 3 | | Unbelted N= 3 |  |  |
|  |  |  |  |  |  |  |  | Thoracic injury | | | RR= 1.22 (0.27, 5.46) | |
|  |  |  |  |  |  |  |  | Belted N=8 | | Unbelted N=3 |  |  |
| **Viano & Parenteau**  **2017**  **NASS-CDS**  **US** | Front | >15 | NR | NR | 17,862,082 | 15,391,527 | 2,905,320 | Head injury | | | RR= 0.49 (0.22, 1.08) | |
|  |  |  |  |  |  |  |  | Belted N= 14,461 | | Unbelted N= 15,011 |  |  |
|  |  |  |  |  |  |  |  | Brainstem injury | | |  |  |
|  |  |  |  |  |  |  |  | N= 2507 | | N= 2,566 |  |  |
|  | Side | >15 | NR | NR | 7,992,145 | 7,612,771 | 958,977 | Head injury | | |  | |
|  |  |  |  |  |  |  |  | N= 21,404 | | N=18,352 |  |  |
|  |  |  |  |  |  |  |  | Brainstem injury | | |  |  |
|  |  |  |  |  |  |  |  | N=3705 | | N=1,759 |  |  |
|  | Rear | >15 | NR | NR | 2,644,179 | 2,587,028 | 234,078 | Head injury | | |  | |
|  |  |  |  |  |  |  |  | N=1,901 | | N=1,957 |  |  |
|  |  |  |  |  |  |  |  | Brainstem injury | | |  |  |
|  |  |  |  |  |  |  |  | N=389 | | N=597 |  |  |
|  | Rollover | >15 | NR | NR | 3,023,982 | 2,615,988 | 647,711 | Head injury | | |  | |
|  |  |  |  |  |  |  |  | N= 9,203 | | N=11,150 |  |  |
|  |  |  |  |  |  |  |  | Brainstem injury | | |  |  |
|  |  |  |  |  |  |  |  | N=1,069 | | N=1,547 |  |  |
|  | All | >15 | NR | NR | 41 596 417 | 33,040,347 | 8,556,070 | Head injury | | | RR= | |
|  |  |  |  |  |  |  |  | N=51,739 | | N=53,382 |  |  |
|  |  |  |  |  |  |  |  | Brainstem injury | | |  |  |
|  |  |  |  |  |  |  |  | N=8,665 | | N=7,770 |  |  |
| **Abbreviations: AIS: Abbreviated Injury Scale; CIREN: Crash Injury Research and Engineering Network; F: female ; M: Male; ; NASS-CDS: National Automotive Sampling System- Crash Worthiness Data System; NCIS National Coronial Information System; NA: Not Applicable; NR: Not reported; NTDB: National Trauma Databank; OCCO: Office of the Chief Coroner of Ontario; USA: United States of America.** | | | | | | | | | | | | |
